# A protocol to build soil descriptions for APSIM simulations

**DOI:** 10.1016/j.mex.2021.101566

**Published:** 2021-11-06

**Authors:** Rogerio Cichota, Iris Vogeler, Joanna Sharp, Kirsten Verburg, Neil Huth, Dean Holzworth, Neal Dalgliesh, Val Snow

**Affiliations:** a The New Zealand Institute for Plant & Food Research Limited, Lincoln, New Zealand; b CSIRO Agriculture and Food, Canberra, ACT, Australia; c CSIRO Agriculture and Food, Toowoomba, Qld, Australia; d AgResearch Limited, Lincoln, New Zealand

**Keywords:** Process-based modelling, Farming systems, Parameterisation, Plant available water capacity, Soil physics, Soil organic matter

## Abstract

Soil processes have a major impact on agroecosystems, controlling water and nutrient cycling, regulating plant growth and losses to the wider environment. Process-based agroecosystem simulation models generally encompass detailed descriptions of the soil, including a wide number of parameters that can be daunting to users with a limited soil science background. In this work we review and present an abridged description of the models used to simulate soil processes in the APSIM (Agricultural Production Systems sIMulator) framework. Such a resource is needed because this information is currently spread over multiple publications and some elements have become outdated. We list and briefly describe the parameters, and establish a protocol with guidelines, for building a soil description for APSIM. This protocol will promote consistency, enhancing the quality of the science done employing APSIM, and provide an easier pathway for new users. This compilation should also be of relevance to users of other models that require detailed soil information.

• This paper presents a brief description of the models for simulating soil processes in the APSIM model.
• The method stablishes guidelines to define the parameters for building a soil description for APSIM.

## Highlights

• Introducing the models and user interface for characterising a soil in APSIM simulations.
• Listing and describing the parameters needed for building soil descriptions in APSIM.
• Providing recommendations for good practice when setting up soil parameters in APSIM.

## Graphic abstract

**Figure:**
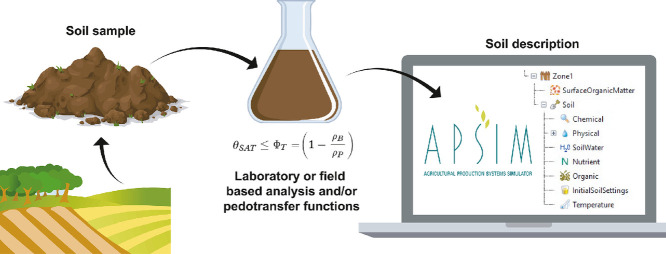

Specification table

**Table:** 

Subject Area:	Environmental Science
More specific subject area:	Agro-ecosystems modelling
Method name:	APSIM soil parameterisation
Reference for original method:	www.apsim.info/documentation/model-documentation/soil-modules-documentation https://www.apsim.info/apsim-model/apsoil Dalgliesh, N.; Hochman, Z.; Huth, N.; & Holzworth, D. 2016. A protocol for the development of APSoil parameter values for use in APSIM - Version 4, CSIRO, Australia. 24 p. Huth, N.I.; Bristow, K.L.; & Verburg, K. 2012. SWIM3: Model use, calibration, and validation. Transactions of the ASABE, 55(4):1303-1313. Probert, M.E.; Dimes, J.P.; Keating, B.A.; Dalal, R.C.; & Strong, W.M. 1998. APSIM's water and nitrogen modules and simulation of the dynamics of water and nitrogen in fallow systems. Agricultural Systems, 56(1):1-28. Verburg, K.; Ross, P.J.; & Bristow, K.L. 1996. SWIMv2.1 user manual. Divisional Report, CSIRO - Division of Soils, Melbourne. 109 p.
Resource availability:	n/a

## Introduction

Computer simulation models are now a common tool for research and are increasingly being employed for decision support [1,2]. There is a wide variety of models available for simulating agricultural systems at different scales and/or different levels of detail. Process-based models are developed with detailed descriptions of the processes in the soil-plant-atmosphere interface and thus are well suited for the investigation of interactions between land use, management practices, and environmental conditions [2], [3], [4]. Such tools can be used for improving our understanding of current farming practices and for testing potential changes under alternative scenarios [2,5]. Models used in this manner can then be employed for optimising management, reducing environmental impacts, or developing mitigation and adaptation practices for climate change [6], [7], [8].

Process-based simulation models of agroecosystems generally encompass detailed descriptions of the soil and the mechanisms which describe soil processes. Soils are crucial for water and nutrient cycling, partially regulating plant growth and discharges to the wider environment [9,10]. Providing appropriate parameters for the characterisation of soils in models is thus important if the results are to be used with confidence [10], [11], [12]. The set of parameters required to describe a soil varies depending on the model, but in any case, parameters should be of good quality. When developing a set of soil parameters it is important to have some understanding of how the model works, how sensitive it is to input data, and which parameters are enduring properties (i.e., are static) or are only initial conditions and are changed by the model throughout the simulation period.

Availability of measured soil data for specific sites is always limited and is seldom sufficiently complete for most process-based models. Thus, it is common that users need to supply inputs that are fine-tuned, or even fully replaced, using some combination of data from similar soils, from nearby locations, from expert opinion, or from pedo-transfer functions (PTFs). Access to soil data for describing soil profiles and improving PTFs is ever increasing, with a few soil databases made available for some regions worldwide, for instance UNSODA [13] and WISE [14]. However, these do not cover all areas, some are not freely available, and generally they are not comprehensive enough to provide all the inputs needed for specific models. PTFs are often based on a limited range of data and generally are most appropriate only for the region or soil types used to develop them [15]. Even when available, such data are likely rather coarse spatially and may need to be adapted to better fit the conditions of a particular site. Modifications based on local knowledge can improve model performance, but can also cause spurious results if the data entered are not appropriate for the model [16,17].

APSIM (Agricultural Production Systems sIMulator [18,19]) is a modelling framework widely used in Australia and New Zealand, and its use is increasing worldwide. It is open source and is free for use in non-commercial applications. New models are added to the framework regularly. Model documentation is now a requisite for the incorporation of a new model into APSIM, but for old, legacy models, such resource is lacking, is outdated, or is spread out over various publications. A primary example of this is the soil characterisation for an APSIM simulation. The documentation and guidelines can be found spread out in older papers and reports [20], [21], [22], [23], plus brief descriptions in websites (www.apsim.info). There is no comprehensive, abridged description of the basic inputs and specific parameters needed by the models used to simulate the various soil processes in the APSIM framework. This is a major hurdle for beginners and users with limited soil science background.

The main objectives of this paper are to review the background and present a description of the various models used in the APSIM framework to simulate the soil and its processes. For each model and inputs, the brief description is followed by methods that can be used to obtain the values needed, with some discussion about their relative importance for the model's outputs. Our purpose is to establish a basic methodology or protocol for setting up soil descriptions for APSIM simulations. With this we seek to ensure consistency and improve the quality of the research in which APSIM simulations are employed. This methodology should also be of relevance to users of other models that require similar detailed soil information.

## APSIM overview

APSIM is an agroecosystem modelling framework for simulating a broad range of agricultural production systems; from annual and perennial cropping, to perennial horticulture, forestry and grazed pastoral systems. It is developed and maintained by the APSIM Initiative (www.apsim.info). APSIM has a large and increasing number of developers and users worldwide; as such, it has been extensively tested in a wide geographical range. A comprehensive list of articles published using the APSIM framework can be found on the APSIM initiative website (www.apsim.info/Products/Publications.aspx). Two main versions of APSIM are currently available, a legacy version (termed here *Classic*) developed and maintained since the 1990’s [19] and a modern version (termed *Next Generation*), with a new code base and where all the current development takes place [18]. The large number of users, plus its modular nature and the fact that its code is open access makes APSIM an invaluable base for research collaboration across disciplines and institutions [24].

Given its modular nature, APSIM consists of several individual models, each representing a component, or subcomponent, of the modelled system (e.g., the weather, the soil, a crop, a plant organ). In the *Classic* version, the models are linked to the core APSIM ‘engine’, which handles the communication between them [19]. In APSIM *Next Generation*, the various models can exchange data directly with each other as all are built using *.Net* language protocols [18,24]. Different models can be added or removed from a simulation to describe different systems (the exchange of crop models being a typical procedure) or to describe the system using different approaches (such as using a simpler model instead of a more complex one). The case of simulating the soil and its processes is an example of the latter, as detailed below.

As viewed using the APSIM interface, an APSIM simulation is a collection of nodes. Each simulation contains at least one Zone (or Paddock) node, which is the basic simulation unit and defines a single point in space (a simulation can have several Zones to account for spatial variation, for instance). Under this node, there are several child nodes, each generally corresponding to a specific model. Several of these nodes have an interface for entering some parameters and/or initialisation values that are used by the model (note that not all models allow setting up parameters via the interface). One of the nodes found within almost all Zones is the soil node, which is one of the fundamental components of an APSIM simulation.

### The soil node in APSIM

**Fig. 1:**
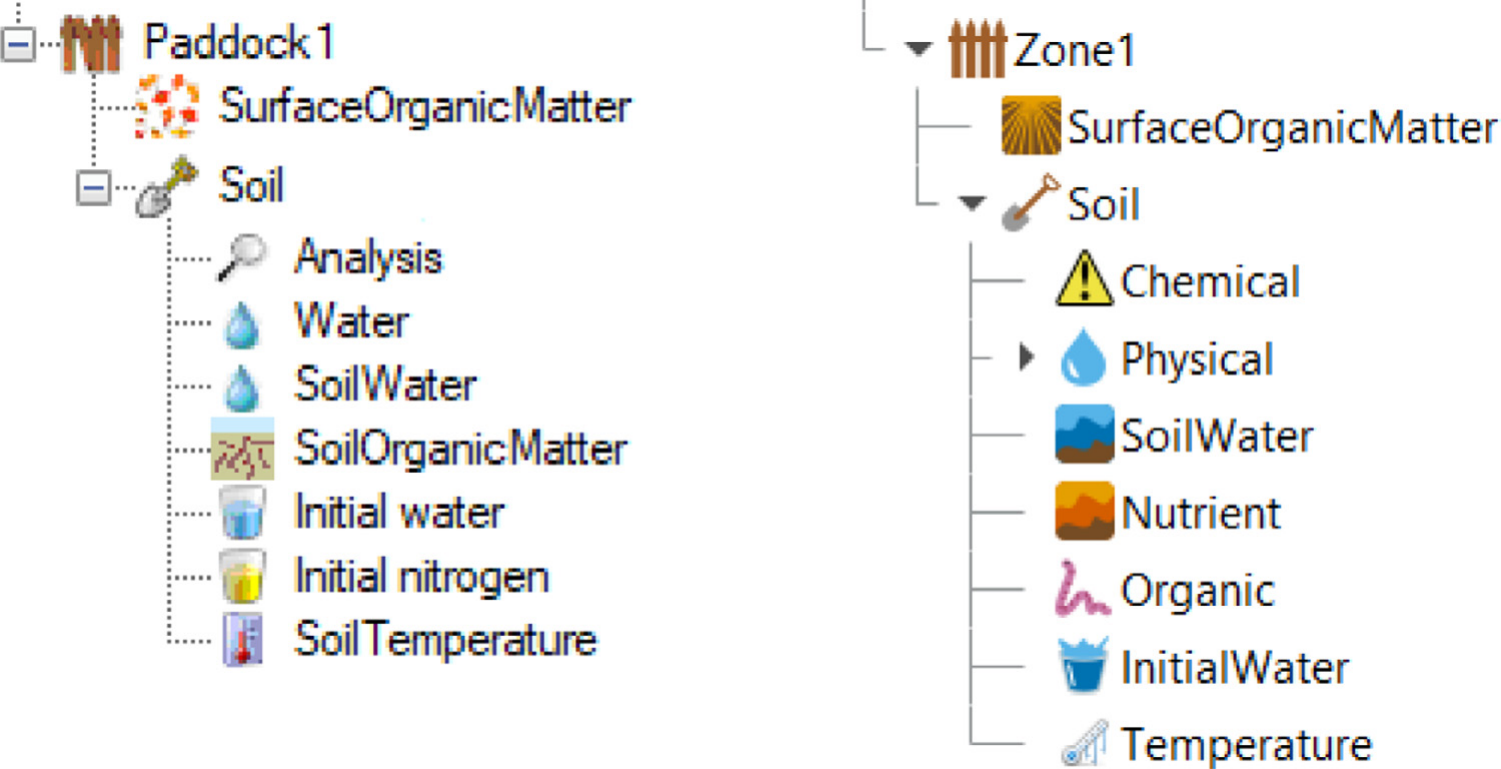
Screenshot of APSIM interface (*Classic*, version 7.10, on the left and *Next Generation*, version 2021.11.10, on the right-hand side) showing the nodes representing the basic spatial point being simulated (a Paddock or Zone) containing a node representing the models for surface residues (SurfaceOrganicMatter) and the soil. The soil node is further expanded to show its child nodes, which can be used to set up the soil properties and the sub-models used to simulate the various processes in the soil (Note: SoilNitrogen is used in APSIM *Classic* but its node is not visible on the user interface).

The soil node is itself a container where several further child nodes define various aspects of the soil and the sub-models used to describe it (Fig. 1). These sub-models account for a set of processes in the soil: soil water movement and solute transport; carbon (C) and nitrogen (N) cycling; and the soil thermal regime. Each model can have variants or alternatives to simulate the respective processes using different approaches. These alternatives differ in the approach and level of complexity used to describe the relevant processes and the model variants can be used interchangeably in most simulations. However, there are situations in which the more advanced capabilities of a particular variant becomes essential. A brief description of the models is given below, this is followed by detailed descriptions of their parameters and how to obtain them, but first a short introduction of the nodes and the variation between the two versions of APSIM is presented.

The child nodes within the soil node comprise entry points for general soil parameterisation (layer structure, physical and chemical properties, initial values for water, organic matter and nitrogen) as well as for setting up model-specific parameters (SoilWater, SoilTemperature, etc.). As part of the continuing development of APSIM *Next Generation*, the soil node was re-structured in 2020. The general overview of the *Classic* and *Next Generation* versions is shown Fig. 1. Although the vast majority of parameters are the same, the differences between the two versions make it difficult to describe the parameterisation process in detail following the node structure. Therefore, we instead describe the general principles for soil parameterisation and the specific recommendations for parameters organised by broad groups (physical properties, soil organic matter, etc.). In this process we will point out differences, if any, between the different versions.

### The SoilWat model

**Fig. 2:**
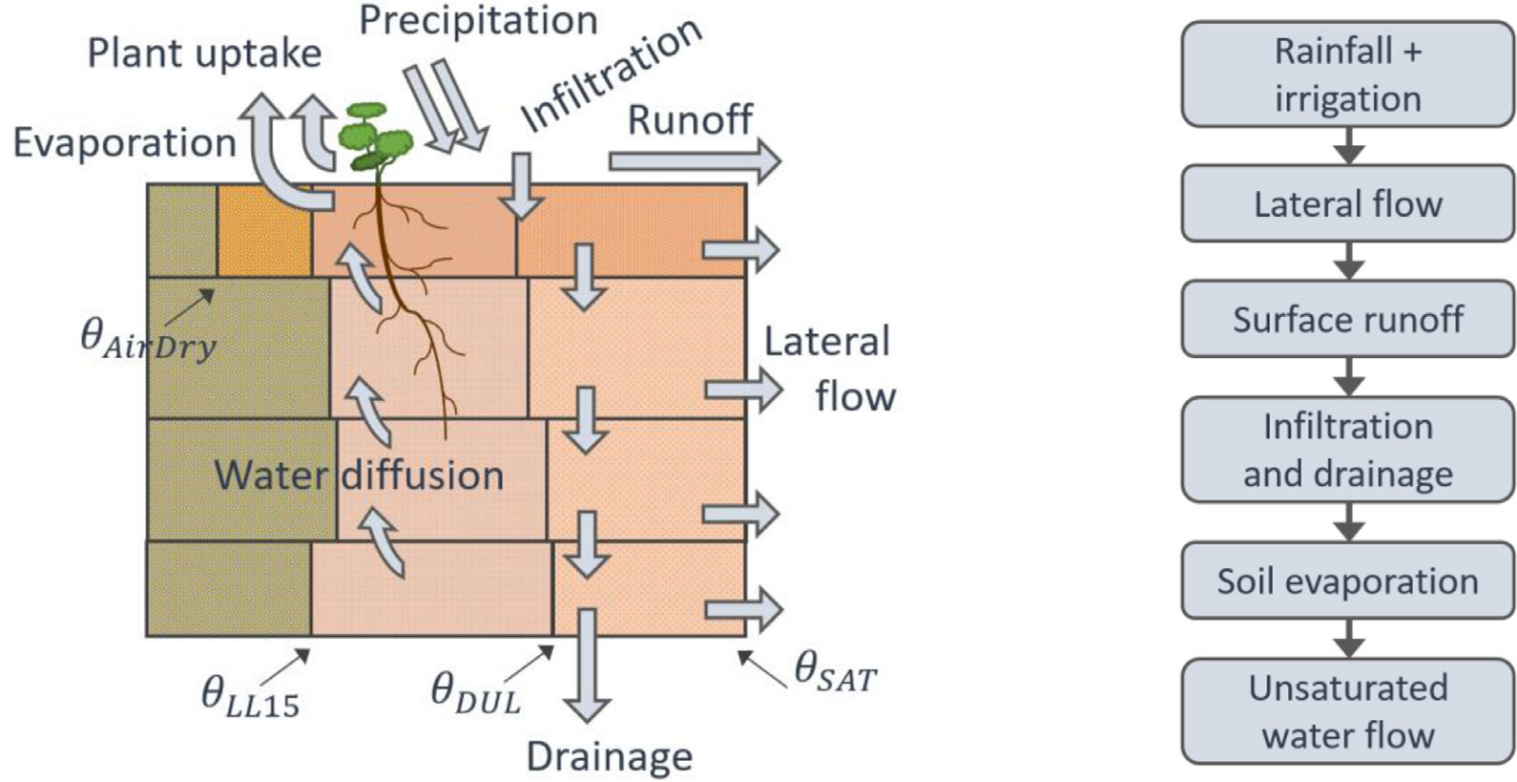
Left-hand side: Schematic of the process involving water movement accounted for by the SoilWat model in APSIM. For each of the four soil layers, the vertical lines represent the water thresholds used to define water storage capacity, the arrows represent the flows and their position gives an indication of the water content range in which they occur (where $\theta_{AirDry}$, $\theta_{LL15}$, $\theta_{DUL}$ and $\theta_{Sat}$ are the volumetric water contents at air dry, lower limit, drained upper limit, and soil saturation, respectively). Right-hand side: Sequence of calculations for each water movement process; note that water uptake is controlled by the plant and thus in not within the sequence. Also, note that there is a parallel sequence for the computations of solute transport.

SoilWat, or SoilWater, is the simplest water model alternative in APSIM. A schematic of the processes accounted for and sequence of calculations is shown in Fig. 2. This model uses a tipping bucket approach [25,26] to describe water movement; with solutes being either mobile (carried by water) or immobile. The SoilWat model is relatively easy to set up and is fast and computationally robust; consequently, it is extensively used in APSIM simulations. The water characteristics of the soil are specified in terms of three thresholds [22]: the lower limit ($\theta_{LL15}$), the drained upper limit ($\theta_{DUL}$), and the saturated ($\theta_{Sat}$) volumetric water contents. Water movement is described using separate algorithms for saturated and unsaturated flows: near-saturated flow occurs when water content is above DUL, a fraction of the water between $\theta_{DUL}$ and $\theta_{Sat}$ drains due to gravity to the layer below [27]; at $\theta_{Sat}$ water will move to the next layer at rate defined by the saturated hydraulic conductivity. In unsaturated flow (below $\theta_{DUL}$) water moves due to the gradient in moisture content between adjacent soil layers. Solute redistribution is computed based on the saturated and unsaturated soil water flows assuming that the water and solutes entering or leaving a layer are completely mixed.

### The SWIM model

SWIM (Soil Water Infiltration and Movement) is a more complex soil water model. It uses a mechanistic approach based on a numerical solution of the Richards’ equation for water movement and the convection-dispersion equation for solute transport [23,28]. The processes accounted for are broadly the same as those for SoilWat (Fig. 2) with the exception of lateral flow, although SWIM does allow for the simulation of artificial drainage [23]. However, the computations are done simultaneously for most of the processes using numerical solutions to the flow equations. SWIM requires a more detailed description of the soil properties (e.g., soil hydraulic functions and solute adsorption isotherms), it is computationally demanding, and can suffer from numerical instability. The SWIM model evolved from a standalone tool into its current form, incorporated within the APSIM framework which comprises two versions: SWIM2 [29], derived from the first integration of the model into APSIM [30,31], and the latest version SWIM3 [21]. Both versions use basically the same procedures to simulate the soil processes, but differ on how the input parameters are set up and some functionality. For SWIM2, the parameters are read by the model from xml files, which need to be pre-compiled – this can be done manually or using tools such as Hyprops [23]. Also, ancillary models, such as SoilNitrogen, have to be explicitly included in the simulations as the soil node itself has to be removed from the APSIM interface (see also Fig. 4, and further description in Section 3.7.2). Dealing with these issues requires considerable expertise, this limits the number of users of SWIM2 but does provide a degree of flexibility not accessible in SWIM3. SWIM3 uses the existing APSIM soil node interface (replacing the SoilWater node with a Swim node, see Figs. 1 and 4). The ability to use the APSIM interface to set up soil parameters directly makes SWIM3 much more accessible to a wide range of users. However, this was accomplished via several simplifications: the hydraulic functions are derived from the three basic soil water thresholds and saturated hydraulic conductivity [21] instead of being defined explicitly using a wide range of functions as is with SWIM2. The capability to describe non-linear processes in the soil is fully available in SWIM3, making it an attractive alternative, particularly where there are complex boundary conditions (e.g., lysimeters, shallow water tables, artificial drainage) or when thin soil surface layers are needed.

### The SoilNitrogen or nutrient model

**Fig. 3:**
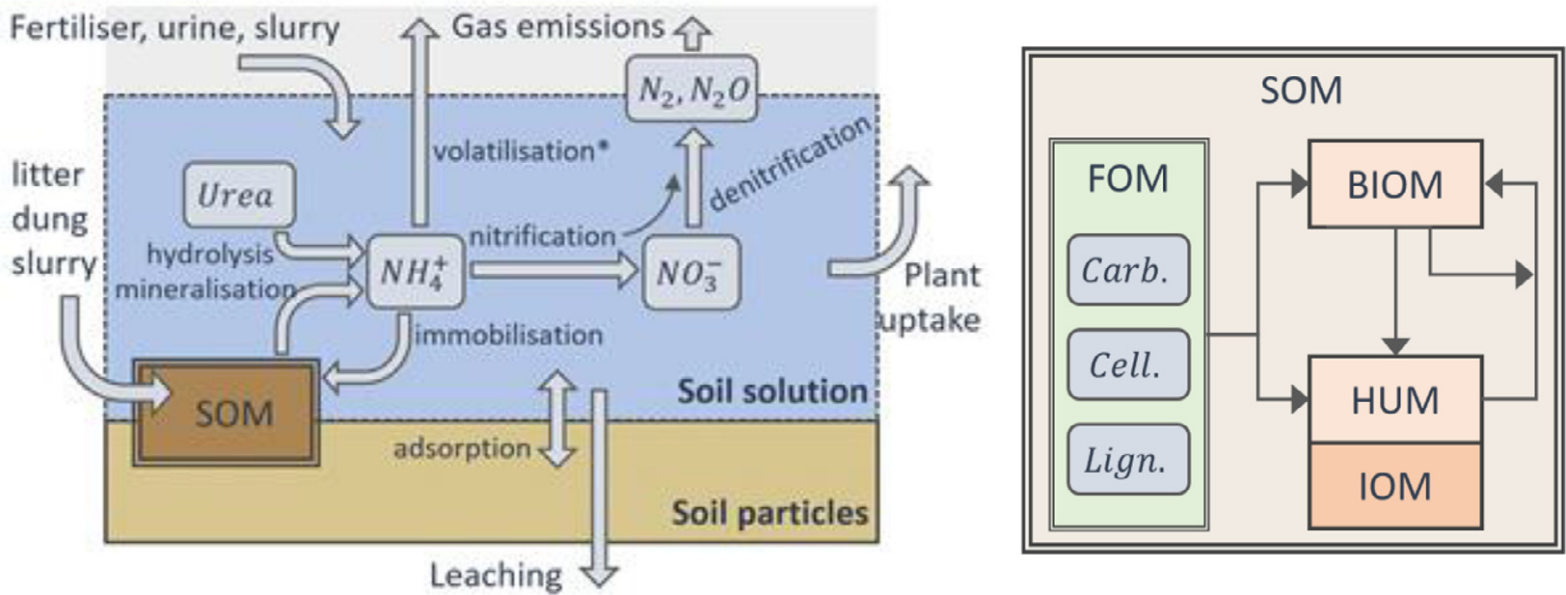
Schematic of the process involving soil N and C cycling in the soil accounted for by APSIM. Mineral N processes are shown on the left-hand side, depicting the three soluble forms and the gaseous forms (note that volatilisation is not included in the release versions of APSIM). On the right-hand side, a more detailed schematic depicting the soil organic matter (SOM) pools and flows is shown (where FOM is the fresh organic matter, comprised of three pools named carbohydrates, *Carb*, cellulose, *Cell*, and lignin, *Lign*; BIOM is the microbial biomass pool, HUM and IOM are the active and inert pool of humic pool, more details about the pools in the text). The arrows represent flows cycling SOM between pools; in each, C can be lost as $CO_{2}$ and mineral N can be consumed (immobilised) or released (mineralised) into the soil solution.

The primary companion of either of the soil water modules is the soil N and C model, SoilNitrogen (SoilN in older literature) or, more recently, Nutrient (available in *Next Generation* only). These models simulate the cycling of C and N between mineral and organic pools in the soil, including processes such as mineralisation and denitrification. Schematics for the main processes accounted for are shown in Fig. 3. Similar to other soil organic matter (SOM) models, such as RothC [32] and CENTURY [33], APSIM's soil carbon and nitrogen model is based on dividing the soil organic material into conceptual pools [22]. These different pools are assumed to follow first-order kinetics, each with its particular turnover rate and efficiency of C retention. Default turnover rates have been determined from experimental and observational data. As the pools are only conceptual and not measurable, individual states and rates for the pools can only be inferred from their lumped behavior. The temperature and moisture modifiers imposed on the decay rates of these pools are typically derived from incubation studies, and assuming that temperature and/or moisture effects are the same for all the different conceptual pools.

The structure of the SOM modelling in APSIM is predominantly inherited from CERES-N [22,34], with some modifications. The main SOM pools include the fresh organic matter pool (FOM), the microbial biomass pool (BIOM or Microbial), and the humus pool (HUM or Humic). The FOM pool represents relatively fresh plant material such as dead roots and crop residues incorporated into the soil. This material is further subdivided into three sub-pools, termed carbohydrate, cellulose, and lignin; each with different C:N ratios and progressively slower turnover rates, allowing for the definition of residues of different quality. The BIOM pool represents fast turnover soil organic material related to microbial biomass and microbial products. The HUM pool represents the bulk of SOM, which is subdivided into an active fraction, subject to decomposition at a slow rate and an inert organic matter fraction (IOM), which is assumed not to be subject to decomposition. Calibration and verification tests for this approach have been undertaken in both CERES [35], [36], [37] and APSIM [7,^38^,39].

In the SoilNitrogen model, a method to simulate within-field spatial variations in the soil carbon and nitrogen cycling has been implemented by Snow et al. [5]. This capability can be useful for simulating systems that have very high spatial variations in nitrogen in the soil. Examples of this include urine patches in grazed paddocks and banded fertiliser applications.

### The SurfaceOrganicMatter model

In addition to SoilN or Nutrient, SurfaceOrganicMatter (also called RESIDUE or SurfaceOM in the past) represents fresh organic matter on the soil surface [22]. Cohorts of above-ground organic residues have mass, C:N, standing fraction and type. Type is selected from a pre-determined list of residue types (such as that derived from wheat, maize, or animal excreta) and is used to determine the overall C fraction, specific area, potential decomposition rate, mineral N content, and C and N fractions representing each of the carbohydrate, cellulose and lignin pools (similar to the soil FOM described above, Fig. 3). The module can track multiple cohorts of surface residues.

Residues decompose on the soil surface according to the potential decomposition rate, temperature, moisture, C:N and contact with the soil surface, and as they do, join the soil OM pools in the layers near the soil surface. Mineral N components can leach into the soil as water infiltrates, and tillage events can redistribute a given fraction of the residues to predetermined soil depths, with residues joining the FOM pools in the corresponding layers. The soil models also interact with the SurfaceOrganicMatter and crop models, so that the simulation of the soil water balance responds to changes in the status of surface residues as well as uptake and crop cover.

### The SoilTemperature model

Temperature affects a variety of processes in the soil and thus needs to be accounted for in APSIM simulations. The default model uses a basic approach whereby the soil temperature is determined from the daily average surface temperature, adjusted by a normalised variation over time (a sinusoidal function) and an exponential function down the soil profile. This is an implementation of the approach in EPIC [40] and requires inputs for the annual average air temperature and amplitude, which are specified in the weather data file. In the *Classic* version of APSIM this is a sub-model of SoilNitrogen and thus is not visible in the user interface. In APSIM *Next Generation*, the model is explicitly shown in the interface. A more comprehensive approach can be used by including the SoilTemperature model (also called SoilTemp in older documentation) to the simulation. This is an implementation of the approach described in Campbell [41] and requires clay content as an input. SoilTemperature is not yet available in *Next Generation*.

## Soil parameters for APSIM

Setting up a soil in an APSIM simulation should, ideally, be based on site-specific measurements. However, field and/or laboratory measurements for all parameters are often not readily available or their determination is impractical. In such cases, estimates based on general information for the location of interest, or the soil type, must be used. When possible, using expert knowledge about the soil can greatly improve its description in the model. A complex model such as APSIM requires a large number of inputs and parameters, but the same information can be used for both versions of APSIM, *Classic* and *Next Generation*. However, some of the parameters are organised differently or have slightly different names in the two versions. A brief description of each parameter that can be set up from the user interface is provided below, along with protocols for the development of the required input information.

It is possible to start setting up a new soil in APSIM from a blank soil node, but the easiest way to set up a new soil is by modifying an existing soil from another simulation or from the APSoil database. APSoil is a repository of soils information developed originally for Australian soils for use in the APSIM framework [20,42]; over the years it has been gradually expanded to include selected soils from New Zealand, USA, Africa and Asia. Modifications can be made to any existing soil and these can then be saved by the users under a new name. Note that soils modified in this way do not enter the public domain as part of the official APSoil database (a protocol [20] to add a soil parameterisation to APSoil can be found at www.apsim.info/apsim-model/apsoil).

### Base soil node interface

The base APSIM interface, displayed when the soil node is selected, presents a set of descriptors for the soil as well as general comments. These can be used to identify the soil type or name, soil texture, location (latitude and longitude in decimal degrees) or address whence the data originated or can be assumed to represent, and information about the data source. Even though most of these data are not used in APSIM calculations, it is important to enter as many details as possible to ensure good documentation. Information about the source of the data and comments about assumptions may be useful for future reference, to help understand how the soil has been described, what data were measured or estimated and so on. In APSIM *Classic* versions, the value defined after “Soil type, texture or other descriptor” is used by SoilNitrogen/SoilN to adjust some of its internal parameters (e.g. turnover rates), but only if that value matches a small set of keywords (by default “sand” or “rothc”, but users can add other variants).

### Layer structure and depth

The depth field appears in several of the child nodes (e.g., SoilWater). These values specify the depth intervals in the soil profile by which the parameters in the respective node are provided. The intervals are commonly set to match natural major variations in soil attributes, i.e., they generally match soil horizons. As the various nodes are independent, the depth field can be different for each node, following the different gradation or available information for a given parameter over the profile (e.g., several horizons may have the same C content and thus can be lumped together for initialisation). The layer structure in which the computations are performed during the simulation can be defined in two ways: APSIM will use the layer structure provided in the soil water balance node (Water, SoilWater, or Swim) but if a child node called LayerStructure is added, then that layering will override all others. During initialisation, APSIM will re-map the parameters provided in all nodes to the final layering, either splitting or averaging layers as appropriate. This is done using a cumulative volume or mass system depending on whether the parameters are volumetric (e.g., volumetric soil water content) or on a soil mass basis (e.g. bulk density)

There is no maximum depth for the soil in APSIM, but often agricultural soil descriptions will not go beyond 1500–2000 mm, depending on data availability. Descriptions for deeper layers may be needed for deep-rooted plants (e.g., lucerne and forestry). If the simulation is to be used for a shorter soil depth (e.g., for a lysimeter experiment), the soil information can be cut off explicitly or the LayerStructure node can be used to define the appropriate maximum depth (but also note the need to define the lower boundary conditions [21,23]). Certain APSIM outputs (e.g., drainage and N leaching) are only given for this depth at the bottom of the profile, although these flows can also be obtained for the different layers.

#### Layering in SoilWat

The water movement in the tipping bucket approach used by this model is dependent on layer thickness (i.e., the same soil properties given at two different layering patterns will give different drainage rates). Generally, thicker layers are specified in this model, so the layer structure can be aligned with soil horizon boundaries. However, it is recommended that the thickness of the layers actually used in the calculation should between 100 and 400 mm. The use of many thin layers can artificially slow down water flow as each ‘bucket tip’ takes one day; whereas using just a few thick layers can cause water to move too fast. The thickness of the top layer is recommended to be 100 to 150 mm, as direct soil water evaporation comes only from this layer. A layer too thick would result in over-estimation of evaporative losses, while a thin layer may underestimate evaporation loss. Furthermore, the topsoil is where some of its properties (e.g., organic matter content) vary the most, so using thinner layers near the surface and thicker at depth allows for a better soil description. Again, note that the layer structures for setting up the soil water and organic matter parameters can be tailored for each node.

#### Layering in SWIM

The layers used in the numerical solution in SWIM must be relatively thin when there are large fluctuations in flow rates or properties with depth or time to avoid numerical instability. This is particularly useful for simulating conditions in which solute concentrations change sharply with depth in the soil (e.g., Cichota et al. [43]). The thickness of each layer will vary from millimetres to a few centimetres. The computation is faster when layers are thicker, but numerical instability is more likely. To ensure convergence of SWIM calculations, it is recommended to use thinner layers close to the surface as well as around sharp horizon transitions. Depending on boundary conditions, the layers at the bottom of the soil might also need to be relatively thin. The solution of soil water flux equations within SWIM may struggle to converge for soils with low hydraulic conductivity, such as soils with high clay contents. In this case, using smaller layer thicknesses reduces numerical instability and increases the likelihood of successful computations. Note that the layering for calculation is generally different than the layers (or horizons) used to provide the soil parameters. In SWIM2 the calculation layering is given as part of the xml input file (e.g. Verburg et al. [23]), while the LayerStructure node is used when SWIM3 is the soil water model. Note that SWIM3 will convert the layer structure into a node structure, which is actually used in the computations [23]. As the number of nodes is the same as the number of layers, some layer structures can result in an unbalanced node structure, so it is useful to check the simulation summary file that the resulting node structure is as intended.

### Soil physical properties

The physical properties of a soil define most of its hydraulic behavior and interactions with the plants. Thus, setting up the best values for these parameters is very important. In APSIM *Classic*, the values are primarily entered in the Water node, with texture and rocks sitting at the Analysis node. In APSIM *Next Generation* all the values are set up through the Physical node. Some additional parameters, specific to the water balance model being used, are entered in the dedicated model node (e.g., SoilWater, Swim).

#### Particle size distribution

Soil texture can be recorded in three fields specifying the percentage composition of the key particle size classes: sand, silt and clay. These values are currently only used directly by APSIM's SoilTemperature model, but it is recommended to record them whenever possible. As the thresholds between the particle size classes depends on the classification system used (e.g., USDA or ISSS), it is also important to record which system is used. The clay content is currently the only value that is used, when the SoilTemperature or Biochar models are included in the simulation. As the size threshold for clay is the same in most soil classification systems, the system used to separate soil particles is not an issue, but if any simulation is modified to include functions that use either silt or sand content, conversion between systems may be necessary (examples of such conversions can be found elsewhere [44,45]). Considering the system used to partition soil particles can be quite important when using PTFs to derive other physical or chemical properties.

#### Rocks content

This field is used to record the volumetric fraction (%) of coarse fragments (generally assumed to be greater 2.0 mm) for each layer of the soil. These values are not currently used by APSIM directly, but it is important to record them, if available, to have the soil properly characterised. As rocks may comprise large portions of the soil that are impervious to roots and inert to most of the soil processes, the presence of rocks has to be taken into consideration when setting up a simulation. In particular, the physical properties can be significantly affected by the presence of rocks [46]. If properties such as bulk density, water retention and soil C and N are determined in the laboratory, they often expressed with respect to the fine-earth fraction; this is, for instance, the case for New Zealand National Soil Database [47,48]. In this case, the values that are expressed on a volumetric basis (bulk density, texture, and the hydraulic thresholds) should be adjusted prior to entering their values in the Water, Physical, or Analysis nodes in APSIM. The adjustment for each measured parameter P can be made using the formula of Bouwer and Rice [49]:

(1) $$P_{adj}=P_{meas}\left(1-f_{Rocks}\right)$$

where $f_{Rocks}$ is the volumetric proportion of rocks in each layer and the subscipts *meas* and *adj* refer to the value measured and that to be entered in APSIM UI, respectively. Eq. (1) assumes that the presence of rocks reduces the volume fraction of the active soil linearly. For hydraulic conductivity this assumption is not valid, as changes in tortuosity are not accounted for. Novák et al. [50] proposed a modification of the approach above to calculate the saturated hydraulic conductivity of the bulk soil ($K_{S,BulkSoil}$) from that of the fine earth fraction ($K_{S,FineEarth}$):

(2) $$K_{S,BulkSoil}=K_{S,FineEarth}\left(1-af_{Rocks}\right)$$

where a is a parameter that incorporates the hydraulic resistance of the rock fragments to water flow as a function of shape, size and orientation of rock fragments. For stones with a diameter of 10 cm, the authors estimated values for this parameter to be 1.2 for loamy sands, 1.1 for sandy loam and loam, and 1.32 for clayey soils [50]. For single stones, the values for a are higher (1.66, 1.32, and 1.93, respectively).

Note that these adjustments are not necessary when the physical parameters have been determined in the field or with techniques that already take the proportion of rocks into account.

#### Bulk density

Values for the soil's bulk density ($\rho_{B}$) are mandatory in APSIM, entered in g/cm^3^, or Mg/m^3^. Such values are typically in the range of 0.8 to 1.7 g/cm^3^ for most natural mineral soils, while smaller values can be found in organic and peat soils. In the model, the values of $\rho_{B}$ are used to convert solute concentrations in the soil (ppm to kg/ha and vice-versa), thus, erroneous values can have an impact on the set up of initial N and C contents. Bulk density is a property routinely measured, but land use and tillage practices can make these values quite variable, especially in layers near the soil surface. For soils with shrink-swell behavior, $\rho_{B}$ can vary significantly with changes in the water content [51]. It is recommended to use $\rho_{B}$ values determined at field capacity, to minimise the problems with later shrinkage or swelling. Thus, whenever possible, the status of the soil and/or the management prior to soil sampling (e.g. whether the soil was tilled or if it was compacted) should be noted on the ‘Comments’ field of the soil node. Corrections to the value of $\rho_{B}$ may then be made based on this by the user. Note that APSIM soil models do not include computations for dynamic changes in soil properties over time or in response to management (e.g. tillage). This can, however, be implemented via a manager script [52], but this procedure is far from trivial. Some Manager [52] scripts, e.g. Biochar [53], can also change the values of bulk density as a function of tillage and rainfall.

When $\rho_{B}$ values are not available, they can be taken from similar soils, preferably nearby and under similar land use, from a database or soil map [14,^54^,55], or be inferred from other available data using PTFs [56], [57], [58]. Users should preferably select PTFs based on the region of their development.

#### Particle density

The density of soil particles ($\rho_{P}$, g/cm^3^) is used to check that the values of saturated water content ($\theta_{SAT}$), or total porosity ($\Phi_{T}$), and $\rho_{B}$ are sensible, following the expression:

(3) $$\theta_{SAT}\leq \Phi_{T}=\left(1-\frac{\rho_{B}}{\rho_{P}}\right)$$

In APSIM *Classic* the value of $\rho_{P}$ was set to 2.65 g/cm^3^ and could not be changed via inputs in the user interface. In APSIM *Next Generation*, $\rho_{P}$ is a field in the Physical node and can be set to the appropriate value, if available. Otherwise, the default value of 2.65 will be used. The value of $\rho_{P}$ can vary substantially in mineral soil (from 2.5 to 2.8 g/cm^3^) depending on mineralogy, clay and organic matter content [59].

#### Water content at saturation

This field contains the values of the saturated water content ($\theta_{Sat}$, cm^3^/cm^3^), which is the maximum amount of water the soil can practically hold (at soil water potential equal to zero). The values of $\theta_{Sat}$ at soil surface should be around 0.5 for most mineral soils free of stones; being smaller for sandy soils (0.4–0.45), considerably larger for volcanic soils (up to 0.65), and reaching values up to 0.9 for peaty soils. Deep soil layers are often more compact and with lower organic matter and tend to present smaller values for $\theta_{Sat}$, but significant variations exist for different soil types. Management practices, such as tillage, animal trampling and machinery traffic, can lead to substantial changes at layers close to the surface. It is therefore important to note the conditions of the soil being parameterised in the ‘Comments’ field of the soil node, and so that appropriate corrections can be made when the simulations differ from the original conditions.

Values for $\theta_{Sat}$ can be determined in the laboratory or from field measurements; they can also be estimated from the total porosity ($\Phi_{T}$) for each horizon (Eq. (3)). The value for $\theta_{Sat}$ is smaller than $\Phi_{T}$ because of entrapped air (which is nearly always present in field conditions). Entrapped air commonly represents 2 to 10% of the total porosity [60,61]. As total porosity is computed from bulk density, $\theta_{Sat}$ and $\rho_{B}$ values are closely related and often $\theta_{Sat}$ is determined based on $\rho_{B}$ in PTFs, with carbon content also often included [62,63]. Dalgliesh and Foale [51] present a methodology to adjust the values of $\rho_{B}$, $\theta_{SAT}$ and $\theta_{DUL}$ for shrink-swell soils as typical calculations involving $\Phi_{T}$ may result $\theta_{SAT}$ being smaller than $\theta_{DUL}$ (see also [64]).

#### Water content at drainage upper limit

The drained upper limit ($\theta_{DUL}$, cm^3^/cm^3^) field represents the soil water content above which drainage due to gravity starts and it is also the upper limit for the definition of plant available water. This threshold is also known as ‘Field Capacity’ and is often defined as the water content retained after gravitational flow becomes negligible [65,66]. In the field, this can be determined by wetting up an area of soil and letting it drain while being covered to avoid evaporation losses. In many soils, the equilibrium at $\theta_{DUL}$ may be reached after 2–5 days, but on heavy soils this can take weeks [51,64]. When drawing from laboratory soil water retention measurements, $\theta_{DUL}$ is generally assigned to a water potential of -0.1 kPa (−100 cm), so some adjustments must be taken when different water potentials are used (values of −0.06 to −0.33 kPa may be used for different soil types and conventions vary internationally). If using a different threshold for $\theta_{DUL}$, this must be well documented to prevent misunderstandings when comparing different soils or if using the value with SWIM . When employing SWIM3 the potential at which $\theta_{DUL}$ is defined can be specified in the user interface.

The value of $\theta_{DUL}$ must be between $\theta_{LL15}$ and $\theta_{Sat}$, but more precise bounds are difficult to establish. In the absence of measured values, data from similar soils can be used, or estimates using published PFTs [62,^67^,68] based on other soil attributes. Checks against any measured moisture data for the site or conditions being simulated are recommended.

#### Water content at lower limit

This field represents the soil moisture values of the lower limit ($\theta_{LL15}$, cm^3^/cm^3^) for water movement and uptake (approximately equivalent to the permanent wilting point, where root length density is non-limiting) and is commonly defined at −1.5 MPa (−15 bar). This value is commonly measured when the soil water retention curve is determined in the laboratory. Note that this value is not the same as the residual soil moisture ($\theta_{R}$) commonly used in models that employ the van Genuchten [69] or Brooks and Corey [70] models for water retention. However, if using those models, the water content at −1.5 MPa of suction can be readily obtained. The −1.5 MPa laboratory measurement does not always correspond with maximum water extraction in the field and can, particularly for clay soils, be higher than observed maximum water extraction in the field [71]. Field measurements of water left in the soil by a crop after a dry season or under a rainout shelter, or the driest soil water contents across a number of seasons, can provide an estimate of $\theta_{LL15}$. However, near the surface, evaporation can lower the water content below that of −1.5 MPa, and at depth the crop may not extract to −1.5MPa due to limitations in root length density or subsoil constraints affecting rooting or root water uptake. The value of $\theta_{LL15}$ for a given soil layer is mostly related to the soil texture, being very small for sandy soils (0-0.1 cm^3^/cm^3^) and reaching values of 0.25-0.35 cm^3^/cm^3^ for clayey soils, although the type of clay and the presence of organic matter can change this considerably. There is also a wide variety of PTFs available for estimating this parameter [11,^62^,^63^,68]. Note that APSIM has crop-specific parameters that enable setting moisture threshold more akin to maximum field extraction (discussed in Section 3.5).

#### Air dry water content

The values of air-dry ($\theta_{A}$, cm^3^/cm^3^) represent the soil moisture that can be reached after evaporation and thus are important in the model only for the surface layers. These values can be measured, although this is not done routinely. As the soil moisture at air dry conditions and −1.5 MPa seem related [41,72], the values for $\theta_{A}$ are typically estimated based on $\theta_{LL15}$. For the top layer, $\theta_{A}$ value is typically set between half and a third of $\theta_{LL15}$, whereas for subsequent layers, it is usually set to 80–90% of $\theta_{LL15}$ above 400 mm (exceptionally down to 600 mm), and equal to $\theta_{LL15}$ below that depth [20,73]. The air-dry values are not used when SWIM is the water model, but it is recommended, for consistency, that these values are specified.

#### Saturated hydraulic conductivity

The hydraulic conductivity of the soil at saturation ($K_{S}$, mm/day) can be supplied in this field. Measured and published values for hydraulic conductivity are expressed in several different units, so care must be taken when specifying $K_{S}$ values. The saturated hydraulic conductivity is one of the most variable soil attributes and consequently is often missing or incomplete in reported datasets. Therefore, values sourced in the literature, derived from PTFs, and/or estimated by experts frequently need to be used. There are many PTFs for hydraulic conductivity [62,^63^,67], but their performance can be poor outside the conditions for which they were developed. In APSIM, the value of $K_{S}$ is optional when using the SoilWat model. If supplied, the model uses $K_{S}$ to limit the water flow at saturation and thus induce surface ponding; if absent, any water above saturation either runs off or drains, bypassing the soil down to a layer that has capacity to store it. For SWIM, the value of $K_{S}$ is used to define the hydraulic conductivity curve in conjunction with $K_{DUL}$ (discussed below). Therefore, values for $K_{S}$ are important to the simulations, controlling water movement not only at but also below saturation.

### Soil chemical properties

APSIM allows recording a number of chemical parameters that are typically measured in a routine soil analysis, i.e. pH, CEC, nutrient content, etc. These can be accessed in the Analysis and Initial nitrogen nodes in APSIM *Classic*, whereas in *Next Generation* they sit primarily in the Chemical node. Several of the values that can be entered in these nodes are not required by any of the commonly used APSIM models. However, they may be important for understanding and helping to characterise the soil type, and some parameters in other nodes may be related to these, even if only indirectly. Specifying these data is general good practice. It is also possible to use non-standard models and/or manager scripts that employ those values. The most relevant parameters are discussed below.

#### Soil pH

Soil pH regulates several processes in the soil, especially those mediated by microbial activity. In APSIM the values for pH are used by the SoilNitrogen/Nutrient model. However, it is important to note that APSIM, using core modules, currently does not simulate changes in pH, meaning that the values set in this field are used throughout the simulated period. So, if the pH is expected to change for a given condition being simulated, the user should assess which value to enter in order to avoid spurious results. Changes in pH can be controlled by Manager [52] script components, such as Biochar [53] and Volatilisation, but these are not in the standard release of APSIM. A neutral value, between 6.0 and 7.0, can be used to make pH non-limiting in processes such as nitrification. For short-term simulations or when pH is expected to be stable over long time, the user can set the values to measured values to suit particular conditions of the system being simulated. The values for pH to be entered should correspond to measurements made in a 1:5 soil to water mixture. In APSIM *Classic*, soil pH measured in a CaCl_2_ solution can be also entered. The value is converted to the default methods internally.

#### CEC

Cation exchange capacity (CEC) is the maximum quantity of total cations that a soil can hold. It can be used as a measure of soil fertility and the capacity to resist leaching losses. These values are routinely measured in soil analyzes and can be recorded in APSIM expressed in cmol+/kg (or meq+/100 g, which is numerically equivalent). In the absence of measured data, general values can be used, or estimates using PTFs [74], [75], [76]. Values for CEC typically range from 3-10 cmol+/kg for sandy soils, 10-25 cmol+/kg for loams, and 25-40 cmol+/kg for clayey soils [74,77]. The variation within each range depends on the type of clay, soil pH, and especially the organic matter content (for instance, in young peaty soils the CEC can be as high as 100 cmol+/kg). CEC is only used by the SoilTemperature model and some Manager scripts (Biochar and Volatilisation), but given its relevance to soil chemistry, it is recommended to add these values to the soil description when available.

#### Soil mineral nitrogen content

Nitrogen supply is generally the major nutritional limiting factor for plant growth, and its cycling is the core of APSIM's soil biochemical models (SoilNitrogen and Nutrient). Initial values for the two main mineral forms (${\text{NH}}_{4}^{+}$ and ${\text{NO}}_{3}^{-}$) should be supplied (either in ppm N or kg N/ha for *Classic*, only in ppm N for *Next Generation*) via Initial nitrogen or Chemical nodes. These can be measurements, if available, or reasonable estimates based on land use and historical fertiliser usage. As these values are highly dynamic, a simulation with the general conditions and management prior to the time of interest can be pre-run to aid estimating appropriate starting values for these inputs, as well for those of initial water content. APSIM also includes urea as a mineral form in its N cycling models, but urea is not included in the initialisation because it is extremely transient.

#### Cl, EC and ESP

Chloride concentration (mg/kg), electric conductivity (EC, dS/m) and exchangeable sodium percentage (ESP, %) are measures linked to soil salinity. These have been regularly recorded and may be used in APSIM simulations for locations where salinity is a concern [78,79]. The recommendation is to record these values in APSIM, if such data are available, but these are of little concern if not simulating saline conditions.

### Soil-plant specific parameters

In APSIM, plants and soil are simulated using separate models, but they interact to account for processes such as water and nutrient uptake, in addition to root senescence which delivers FOM to the soil. Although most of the interaction is through exchange of information, there are a few parameters that need to be set at initialisation to define how roots grow and interact with the soil. Three parameters are set through the soil node: CLL, KL, and XF (details given below); these are given for each soil layer, thus the soil depth and characteristics as well as the rooting depth for each plant need to be taken into account. As these parameters control the plant-soil interactions, their values matter only within the root zone. Maximum rooting depth can be quite variable, depending on the plant, cultivar and growing conditions, and specific information is not commonly available. Note that the actual depth reached by roots should be an emergent property. Published values for a selection of plants and condition are available. In New Zealand the Foundation for Arable Research provides general recommendation for crops [80], while some information for common pastures species is also available [81]. Internationally, general tables for maximum rooting depths for a selection of plants have also been compiled [82,83]. It is important to note that the soil can limit root growth due to a variety of factors (compacted soil layer, low oxygen status, fragipan or bedrock, high water table, etc.) and these need to be considered when defining the soil-plant parameters. Several soil databases provide estimates of maximum rooting depth from the soil point of view, e.g. Wilde [48] and Malone and Searle [84].

#### Crop lower limit, θ_CLL_

The values of $\theta_{CLL}$ define the lower limit for water extraction by plants. The values can be determined in field measurements in seasons with a dry finish or under a rainout shelter [20]. While many such field characterisations have been performed in Australia and collated into the APSoil database [42], such values are not commonly available elsewhere. Where laboratory $\theta_{LL15}$ are available, the crop's $\theta_{CLL}$ values can be set equal to those of $\theta_{LL15}$. However, it can be useful to set $\theta_{CLL}$ to values higher than $\theta_{LL15}$ for soil layers in which there are only a few roots (towards the bottom of the root zone or where subsoil constraints affect rooting). This can also be used to simulate plants that are more sensitive to drought. Alternatively, drought-tolerant plant may have the capacity to extract water below 1500 kPa, thus $\theta_{CLL}$ can be set to a value lower than $\theta_{LL15}$. These changes must be done with care and taking into consideration the plant being simulated. The crop's $\theta_{CLL}$ can also be set equal to the lowest water contents achieved under a deep-rooted, perennial crop, possibly with small adjustments, as outlined above, to reflect differences in rooting depth and patterns.

#### KL

KL values represent the maximum fraction of the available water that a given plant can take up within one day from a soil layer in the root zone. This is an empirical factor that conflates the effects of soil hydraulic properties (i.e., water conductance towards the root surface) and plant root characteristics (i.e. root length density and root water potential activity). This is a key parameter for estimating water uptake, which unfortunately varies with soil type, depth, as well as crop species and growth stage. Ideally, its value should be determined in experiments, but the procedure is time-consuming and data from such sources are rare. Tables with general recommendations can be found [20] and functions for its variation with depth have been proposed [85]. The later publication assumes a base value near the surface and an exponential decrease with depth, which agrees well with water extraction pattern for the plants tested [86,87] and is likely related to a decrease in root density. Values at the top soil layer vary between 0.06 and about 0.10 [20,85]. Further modifications to this distribution due to soil factors (e.g., a reduction due to a compacted layer or due to constraints like subsoil acidity or salinity) may be also needed.

#### XF

The value of XF, or root exploration factor, represent a simple empirical factor limiting root growth in any soil layer. This restriction can be due to physical constraints, such as a highly compacted layer, or chemical, such as salinity or unfavourable pH. In general, the value of XF should be set to one (full exploration) for all layers, unless soil data suggest that root penetration would be limited. Fully impeding layers, such as bedrock, should have an XF value of zero. Note that this parameter does not affect uptake processes directly, i.e., the function for water extraction in the model does not use this parameter. However, delays in root growth due to a small XF value will likely result in less uptake and thus some result is growth penalty.

Where a crop's $\theta_{CLL}$ profile is chosen equal to $\theta_{LL15}$, the values of KL and XF can be varied to reflect the rooting depth and water extraction patterns of that crop or its sensitivity to subsoil constraints. This is an alternative to adjustments in the crop's $\theta_{CLL}$ [88,89].

#### Soil organic matter

As introduced in Section 2.4, soil organic matter is described in APSIM using conceptual pools [22]. The initial set-up for these, which include both C and N, is made in the SoilOrganicMatter node in the *Classic* version and in the Organic node in APSIM *Next Generation*. The parameters can be best described in two groups, one pertaining to fresh organic material and another for the material that is already decomposed and incorporated into soil organic matter.

#### Fresh organic matter

The fresh organic material (FOM) is primarily derived from recently dead plants (e.g., roots). In APSIM *Classic* a single dry matter amount (kg/ha) and C:N ratio are required (termed ‘Root weight’ and ‘Root C:N ratio’ in the user interface). The amount is distributed down the profile using an exponential function, decreasing with depth; the carbon fraction (set to be 40% of dry matter) and the given C:N ratio of this FOM are assumed to be constant over the soil profile. In APSIM *Next Generation*, the amounts are provided for each soil layer in a field appropriately named ‘FOM’, the C:N ratio of FOM is also needed and is assumed constant throughout the profile (the fraction of carbon in FOM also remains constant at 40%). The amount of FOM varies widely in the field due to land use and management, and unfortunately is seldom determined in routine measurements (soil samples are typically sieved before analysis and larger organic materials such as roots are removed). Amounts of FOM can be large for pastures recently sprayed out using herbicide or after incorporating surface organic matter via tillage, whereas soils or fields where plant material is consistently removed will have lower FOM. Similarly, the C:N ratio can vary greatly, being relatively low from freshly sprayed-out plants (e.g., 20 for grass) to very high for end-of-cycle cereal residues (e.g., 100). The FOM content in the soil can vary quickly over time, depending on its composition and environmental conditions; user discretion is required to set these values appropriately. A good procedure to determine initial FOM values is to run spin-up simulations using the land use that would be on the simulated site prior to the time of interest, the outputs from this simulation can be used to initialise the main simulation; alternatively both simulations can be combined and the early outputs are simply discarded.

#### Organic carbon fractions

Organic matter in APSIM is set up by first defining the content of organic carbon (OC, % or g/100 g of dry soil) for each soil layer. This encompasses all carbon from material that is in the soil excluding fresh material (FOM). Important to note that the term soil organic matter (SOM) is more commonly used in some regions, this represents the total dry matter amount, not only carbon in the soil (their relationship is OC = SOM/1.72, based on an organic C content of 58% [90]). A corresponding C:N ratio is needed to define the amount of N in each soil layer. In APSIM *Classic* the C:N ratio is assumed to be constant over the profile. Both OC and C:N ratio can vary significantly depending on soil type and especially land use, therefore the values to be used in APSIM should preferably come from measurements. Alternatively, values from similar soils and/or literature may be used, but the user must check that they come from conditions similar to those in the simulation. An alternative, or complement, is to do a spin-up simulation using historical conditions prior to those of the period of main simulation, results from this can be then used to establish the likely OC content and distribution over the profile. Care must be taken, however, as OC contents may take several decades to reach equilibrium; also, there are conditions in which it is not reasonable to expect equilibrium and thus it will be difficult to determine when the pre-run should stop, especially if the initial state is too divergent.

In New Zealand, surface mineral soils can have OC of up to 10–15% (volcanic soils under pasture), but commonly found values are 4–5% for pastures and 2–3% for cropping [91,92]. These values reflect the condition of the land and especially its temperate climate. OC values for agricultural soils in Australia, with its drier and warmer climate, are generally much lower. A large study from the Australian National Soil Carbon Research Program (SCaRP) obtained an average of 1.4% across 19,766 samples in the top 30 cm of the soil [93]. The range of values in international data is similar to those above, e.g., Gray et al. [94]. The values of OC decrease quickly with depth, following an approximately exponential function that matches typical root density distributions. The C:N ratio of SOM varies less, being generally between 11 and 12, commonly increasing with depth. Organic and peaty soils can have very high OC values throughout the soil profile [95].

To complement the initialisation of soil organic matter, APSIM requires the values of FBiom, the fraction of organic matter that has fast turnover rates, and FInert, the fraction of humic organic matter that is inert [22]. The fast turnover pool (BIOM) is generally referred to as microbial biomass (having a set C:N ratio of 8), although it is merely conceptual and not explicitly linked to the measurable microbial biomass. Its value is linked to the continuous availability of fresh organic matter and often decreases sharply with depth. Experience in New Zealand and Australia suggest this fraction is around 0.06–0.08 at the surface for non-tilled, good quality soils with high organic matter content, and 0.03–0.04 for intensely tilled or impoverished soils. For deeper layers, at or below the root zone, a value of 0.01 is commonly used as this value reflects a steady-state matching of the ratio of BIOM and HUM pool turn-over coefficients [22]. As this pool has a fast turnover, and is influenced by environmental conditions as well as plant residues, a spin-up simulation can be used to improve the estimates of initial values for FBiom.

The inert pool (IOM) generally increases with depth, reaching nearly 100% near the bottom and of the root zone, where fresh organic matter inputs are minimal; its extent can also vary with soil type and land use. The inert pool represents the carbon content that would result if the soil was allowed to equilibrate over a very long period of time with no addition of fresh organic matter [22], which is equivalent to the Bartholomew and Kirkham [96] equilibrium. In Australia, charcoal content has been used to estimate the IOM near the soil surface, but this is not recommended for deeper layers or in regions where charcoal is not a prominent fraction or cause of inertness in the soil organic matter. Alternatively, OC measured below the bulk of the root system (at about 600 mm for most crops) is a good rule-of-thumb estimate of IOM at the surface. Note that in many models, a pool with a very slow turnover rate is used instead of an inert fraction, whereas in APSIM it is assumed that this pool is fully inert. The remainder of the OC, after the proportions FBiom and FInert are accounted for, is considered to be the HUM pool. When parameterising the organic carbon fractions, it is important to consider the timescale over which the simulation will be run, as the assumptions inherent in the structure of the SOM, such as fixed C:N of the BIOM pool in addition to the fully inert IOM pool, may not be true over very long time periods. As such, additional care and critical examination of the model's outputs is recommended to ensure their behavior over time is sensible.

### Model-specific parameters

A few child nodes within the soil node in APSIM correspond to specific models and provide entry points for their parameters. Given its open-source nature and continuous improvements, there are a number of models that have been (or are being) developed to modify, improve, or test different approaches to simulate soil processes. Only the most commonly used are discussed here, and consultation to their documentation and related publications is recommended (most can be accessed at www.apsim.info).

#### SoilWater node

The presence of this node indicates that the SoilWat model is being used to describe water balance and solute transport in APSIM. The node enables setting up a number of parameters *via* the user interface, the relevant ones are discussed.

##### SWCON

This parameter specifies the proportion of water between $\theta_{Sat}$ and $\theta_{DUL}$ that can drain from each soil layer per day, and originally defined in CERES [22,27]. The value is thus linked to the soil hydraulic conductivity, but also depends on near-saturation water storage ($\theta_{Sat}$ – $\theta_{Dul}$) and layer thickness [97,98]. Measurements for this parameter are costly and time-consuming and thus data are seldom available. Generic values have been suggested for a few soils textural classes (e.g. 0.3 for clayey and 0.8 for sandy soils [20]), but expert judgment is often required to adapt these for specific soils. The use of PTFs or functions derived from water flow theory can also be used to determine this value [26,^98^,99].

##### K_LAT_

This represents the lateral (horizontal) hydraulic conductivity (mm/day) of each soil layer. If set to a value above zero, APSIM will compute the net proportion of water above DUL that is lost laterally from the soil (i.e., removed from the simulation). By default, this value is left empty or set to zero, which means no lateral flow is computed. If such a process is relevant, the user must supply estimates for this parameter, which can be measured or derived from vertical hydraulic conductivity. For many soils the horizontal conductivity can be assumed to be equal to the vertical value [100,101]. Note that if lateral flow is to be computed, a few additional parameters must also be set up: slope gradient (the tangent of the mean slope angle of the field, m/m), discharge width (width of downslope flow boundary, m), and catchment area (above the discharge point, m^2^). Note that in APSIM *Next Generation* slope is a property of the field or zone, rather than being set in the soil node.

##### Soil water diffusivity

The movement of water when soil moisture is below DUL is simulated in SoilWat using a normalised water diffusion approach [22,27]. This process can move water up or downwards depending on the moisture gradient between adjacent layers. Two parameters are used to compute soil water diffusivity: a diffusivity constant ($D_{wc}$) and a slope coefficient ($s_{diff}$) governing the variation of water diffusion as a function of water storage above DUL. Default values in APSIM are 88 and 35.4, respectively, based on Ritchie [27]. For cracking clay soils, 40 and 16 have been found to be more appropriate. For deep sands, values of 250 for $D_{wc}$ and 22 for $s_{diff}$ have been used [20]. As only one set of values is used for the whole profile, the values 40 and16 of the clay subsoils should be used for texture-contrast soils [20].

##### Albedo and evaporation parameters

The albedo number represents the fraction of the incoming radiation that is reflected by the bare soil and is an important component of the energy balance in the calculation of evaporation. This parameter varies considerably depending on the soil type, surface roughness, organic matter content, and soil moisture [27,^102^,103]. Typically, the value ranges between 0.12 for wet soils and 0.35 for dry soils. Note that the albedo of bare soil is fixed for a given simulation, but residues and plant cover can alter the overall surface albedo.

Soil water evaporation in SoilWat is based on the two-stage model developed by Ritchie [104]. The implementation in APSIM only removes water from the surface soil layer, although upwards movement due to moisture gradient can result in the drying of the sub-soil. The evaporation model requires two parameters: U and CONA, which can be supplied separately for winter and summer conditions. The value of U (mm) defines the upper limit of Stage 1 evaporation (i.e., before soil water supply limits evaporative losses), and depends on soil type and environmental conditions [105,106]. The parameter CONA (mm d^−0.5^) controls the water losses in the subsequent Stage 2 and also is dependent on climate and soil type [105,107]. Monitoring of soil moisture in rain-out shelter conditions can be used to determine the values for these parameters, but such data are seldom available. Values in APSoil, which are derived from data and/or expert opinion [20], can be used to parameterise similar soils, but verification against local data is advised. There are also a few publications that explore the variation of these parameters due to soil type and environmental conditions [105], [106], [107]. Both parameters can vary due to climatic conditions, being generally smaller for colder weather conditions (high latitudes and over winter). The value of U can be estimated to be as low as 1 mm in winter and as high as 15 mm for medium textured soils in summer [105,106]. The value of CONA varies less, between 1 and 6 mm/day^2^ [105,107].

##### Runoff parameters

Net runoff amounts are computed in SoilWat using the USDA-Soil Conservation Service procedure known as the curve number (CN) approach [108,109]. This procedure uses total daily precipitation, not taking into account duration or intensity (note that irrigation is by default assumed to be at low intensity and not included in the calculations of runoff, but this can be changed when using the Irrigation model in APSIM by setting the parameter ‘will_runoff’ to 1). Runoff response curves (runoff as a function of total daily rainfall) are specified by CN numbers from 0 (no runoff, water that cannot infiltrate ponds on the soil surface) to 100 (all water that cannot infiltrate will run off). SoilWat accounts for variations in CN due to changes in soil cover, with three parameters given in the interface for controlling these changes. The first value is the CN for bare soil (which typically varies from about 60 to 100), the second defines by how much soil cover reduces the CN value (typically 10 to 30), and the third parameter defines the cover at which the maximum reduction of CN is reached (0.8 it a typical value). Tables with values of CN for a range of covers and soil types are available in the literature [20,109].

#### Swim node

**Fig. 4:**
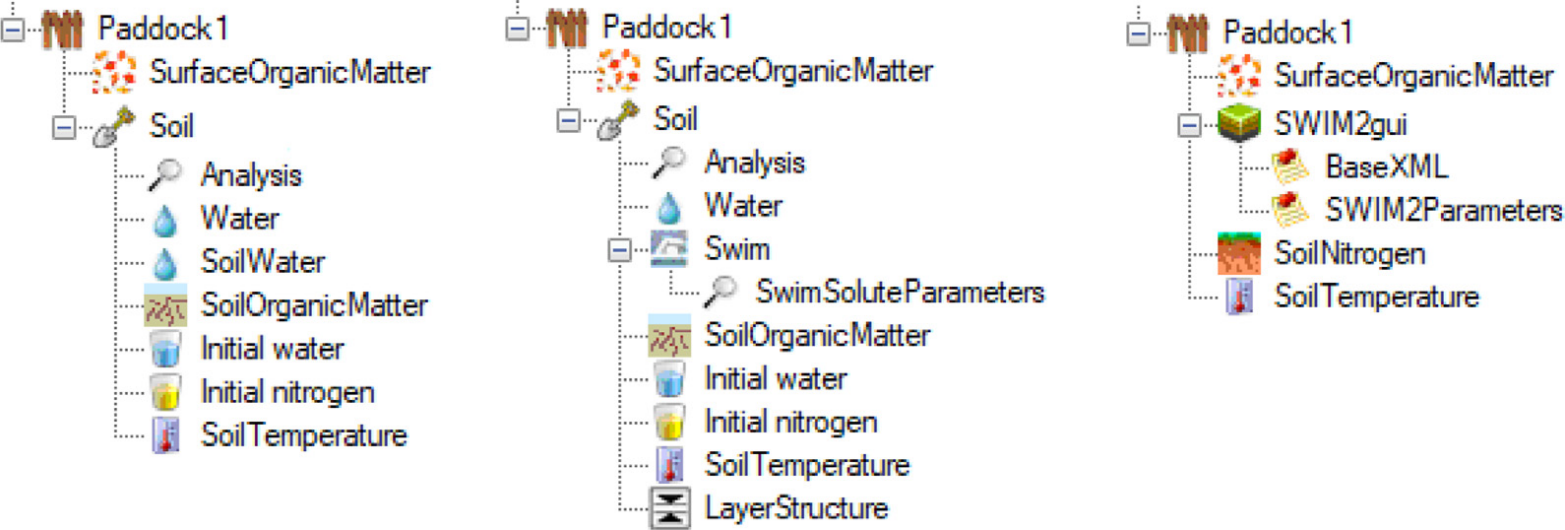
Screenshot of APSIM interface (*Classic*, version 7.10) showing different configuration for the soil node, in the left using the SoilWat model, in the centre using SWIM3 (SoilWater node replaced by Swim, plus LayerStructure), and in the right using SWIM2 (the entire soil node is replaced by SWIM2gui, with SoilNitrogen and SoilTemperature being explicitly added too. The sub-nodes under SWIM2gui are linked to xml files with the basic model- and soil-specific parameters).

Replacing the SoilWater node by Swim, indicates that the SWIM3 model will be used to compute water movement and solute transport. This node can have child nodes for additional functionality (e.g., SwimWaterTable), with SwimSoluteParameters being obligatory. In the Swim node, the user can set up basic parameters used by SWIM to define how the numeric solution will be run as well as a few parameters to aid defining the hydraulic conductivity curve. In APSIM *Classic* SWIM2 can be used, in this case the whole soil node is replaced by nodes representing SWIM2, SoilNitrogen, and SoilTemperature (Fig. 4), with most of the parameters supplied in an xml file. This arrangement requires a high level of expertise to set up, is rather tedious, and so is not commonly used. The parameters required for SWIM2 are largely the same as those for SWIM3, with some exceptions due to changes in some functionality (e.g., runoff computation).

##### Parameters for the numeric solution

These are a series of parameters that can influence the way SWIM makes its calculations [23]. They are generally not modified, unless the calculations do not converge, or at discretion of the user (requiring some level of expertise). These are: the minimum and maximum time-steps, the maximum water increment, and the space weighting factors for water and solute. In case of non-convergence, reducing the maximum time-step and especially reducing the value of water increment generally increases the chance of convergence (at the cost of increased computational time). The space weighting factors (one for water movement and the other for solute transport) also affect convergence of the numeric solution, especially when there is numeric instability. The default value of zero means that SWIM decides if the computations will use central weighting (which can be defined by setting the factor to 0.5) or forward weighting (defined by specifying the value equal to one). Central weighting tends to be more unstable, but produces fewer numerical errors. See Verburg et al. [23] for details.

##### Runoff parameters

For SWIM3, the parameters are the same as discussed above for the SoilWater node, as the runoff routine was adapted to use the CurveNumber approach [21]. In SWIM2 there are options to set runoff to zero, to remove all excess water from the soil surface as runoff, or to use of a simple runoff model [23]. This model assumes that the soil surface roughness detains water that does not infiltrate in a given time-step. This water storage, which is usually of a magnitude of a few millimetres, is related to the height of surface roughness and to the mean slope of the field [23,110]. Water infiltration can be left to be controlled by the soil hydraulic characteristics or additionally limited by surface conductance. Using surface conductance enables accounting for phenomena like surface sealing and water repellence. Both the roughness and the conductance of the soil surface can be set to vary over time; these are related to rainfall events by a power-law function [23,111]. The model computes net runoff rate based on the Manning equation, using the water in excess of surface storage, the gradient and length of the slope, and Manning's number (which vary between 0.016 for smooth clean surface, 0.03 for more irregular surfaces, to 0.15 in vegetated surfaces) [23,112].

##### K_DUL_ and matric potential for K_DUL_

These two parameters are only required by SWIM3 to help define, alongside the saturated hydraulic conductivity, the soil hydraulic conductivity curve [21]. They represent the hydraulic conductivity ($K_{DUL}$, mm/day) and the matric potential (kPa) of the soil at drainage upper limit, or field capacity. These values should be in agreement (i.e., coordination between matric potential and soil water content) with the values specified in the $\theta_{DUL}$ field of the Water or Physical node. A conductivity value of about 0.01 mm/day is typically used in the literature to define DUL [113,114], which implies that the matric potential for DUL varies with soil type. Vogeler et al. [115] estimated, based on soil morphology, that when assuming a matric potential of 10 kPa for $K_{DUL}$, its value ranges from 1 to 5 mm/day in well-drained soils, for moderately well-drained soils it ranges from 0.1 to 0.5 mm/day, and between 0.05 to 0.1 mm/day for poorly drained soils.

#### SWIMSoluteParameters node

This child node of the Swim node allows setting up the basic parameters for the computation of solute transport. In SWIM, solute transport is simulated using the convection-dispersion equation approach [23] and thus require parameters for solute dispersion and adsorption. The concentration of various solute in the water table can also be given in SWIM sub-nodes.

The parameters controlling dispersion can be set by the user, but it is recommended that only those with a good level of expertise do so. The value of dispersivity can be variable enough (typically between 1 and 20) to require attention during setup. Dispersivity values have been related to the hydraulic properties of soils [116,117] and generally are larger for clayey soil. Solute adsorption is described using the Freundlich isotherm [118], which requires two parameters: the exchange coefficient (EXCO) and a power factor (FIP). An EXCO equal to zero means no adsorption, which is commonly assumed for urea and ${\text{NO}}_{3}^{-}$; linear adsorption is defined by setting FIP to one. In general, these values only need to be modified for ${\text{NH}}_{4}^{+}$, unless there is evidence of adsorption of any of the other simulated solutes in a particular soil. In the absence of measured values, the parameters for ${\text{NH}}_{4}^{+}$ can be estimated based on PTFs (e.g. Vogeler et al. [119] for New Zealand soils). As these PTFs have been derived from batch adsorption studies, sorption equilibrium is implied. This can result in overestimation in non-equilibrium conditions. In this case, it is recommended to reduce the value of EXCO based on expert knowledge.

#### SoilNitrogen/Nutrient nodes

The node representing the C and N cycling model is shown explicitly in APSIM *Next Generation*, with SoilNitrogen being replaced by Nutrient as part of the upgrade of APSIM to new model development approaches [24,120]. In APSIM *Classic* the SoilNitrogen model (or SoilN in older versions) was used, but no explicit node in the interface was shown (except when using SWIM2). In all versions, no parameters are exposed to the user in the interface. However, they can be changed by modifying the xml field in APSIM *Classic* or via manager scripts in *Next Generation*, but this requires expert knowledge and describing it is beyond the scope of this work.

#### SoilTemperature/temperature nodes

The soil thermal regime is, by default, computed by the Temperature model in both APSIM *Next Generation* as well as in *Classic* (this was a sub model of the SoilN/SoilNitrogen in older versions). This default model uses a simple approach adapted from EPIC and CERES [22,34] and can be replaced by the process-based SoilTemperature model for more sophisticated simulations (see www.apsim.info, also Chauhan et al. [121]). The simple model does not require any direct parameter input, but uses the average annual air temperature and amplitude, which are specified in the weather data file (in APSIM *Classic* the tool tav_amp.exe may be used to compute these values). SoilTemperature allows several parameters to be modified *via* the user interface, but its parameters are generally not modified and are not described here. It is noted that SoilTemperature requires values for the clay content and CEC over the soil profile, emphasising the need to fill in as many of the soil physical and chemical properties as possible.

## Conclusion

In this work we have reviewed the background and presented descriptions of the models used to simulate soil processes in the APSIM framework. Abridged descriptions of the input parameters for each model have been given alongside a brief discussion on how to set up their values. With these we have established a basic protocol for setting up a soil for APSIM simulations, providing guidelines for beginners and users with limited soil science background. The overarching aim is to promote consistency and improve the quality of the science in which APSIM simulations are employed.

## Declaration of Competing Interest

The authors confirm that there are no conflicts of interest.
